# Supplementary material for: Unveiling the Bioactive Potential of the Invasive Jellyfish Phyllorhiza punctata Through Integrative Transcriptomic and Proteomic Analyses
Source: Biomolecules. 2025 Aug 4;15(8):1121. doi: 10.3390/biom15081121 (PMC12383608; doi:10.3390/biom15081121)
Supplement: Supplementary file 1 [file biomolecules-15-01121-s001.zip › Suplementary Material/FileS3/PEPFOLD4/XP_065066789_5331/clusters.html]

 PEP-FOLD clusters


Download archive of all models  here   

|  |  |  |
| --- | --- | --- |
| # | file | sOPEP |
| # cluster 1 - Size: 25 |  |  |
|  | XP\_065066789\_5331-model1 | -14.9772 |
|  | XP\_065066789\_5331-model1.1 | -14.9772 |
|  | XP\_065066789\_5331-model1.2 | -14.9656 |
|  | XP\_065066789\_5331-model1.3 | -14.9656 |
|  | XP\_065066789\_5331-model1.4 | -14.9412 |
|  | XP\_065066789\_5331-model1.5 | -14.9213 |
|  | XP\_065066789\_5331-model1.6 | -14.9213 |
|  | XP\_065066789\_5331-model1.7 | -14.9213 |
|  | XP\_065066789\_5331-model1.8 | -14.9213 |
|  | XP\_065066789\_5331-model1.9 | -14.9213 |
|  | XP\_065066789\_5331-model1.10 | -14.9213 |
|  | XP\_065066789\_5331-model1.11 | -14.8565 |
|  | XP\_065066789\_5331-model1.12 | -14.8297 |
|  | XP\_065066789\_5331-model1.13 | -14.8297 |
|  | XP\_065066789\_5331-model1.14 | -14.8101 |
|  | XP\_065066789\_5331-model1.15 | -14.7917 |
|  | XP\_065066789\_5331-model1.16 | -14.7917 |
|  | XP\_065066789\_5331-model1.17 | -14.7439 |
|  | XP\_065066789\_5331-model1.18 | -14.7439 |
|  | XP\_065066789\_5331-model1.19 | -14.7439 |
|  | XP\_065066789\_5331-model1.20 | -14.7328 |
|  | XP\_065066789\_5331-model1.21 | -14.7328 |
|  | XP\_065066789\_5331-model1.22 | -14.7328 |
|  | XP\_065066789\_5331-model1.23 | -14.7085 |
|  | XP\_065066789\_5331-model1.24 | -14.6563 |
| # cluster 2 - Size: 17 |  |  |
|  | XP\_065066789\_5331-model2 | -14.9287 |
|  | XP\_065066789\_5331-model2.1 | -14.9287 |
|  | XP\_065066789\_5331-model2.2 | -14.9287 |
|  | XP\_065066789\_5331-model2.3 | -14.8868 |
|  | XP\_065066789\_5331-model2.4 | -14.8387 |
|  | XP\_065066789\_5331-model2.5 | -14.8387 |
|  | XP\_065066789\_5331-model2.6 | -14.8387 |
|  | XP\_065066789\_5331-model2.7 | -14.8387 |
|  | XP\_065066789\_5331-model2.8 | -14.8387 |
|  | XP\_065066789\_5331-model2.9 | -14.8217 |
|  | XP\_065066789\_5331-model2.10 | -14.786 |
|  | XP\_065066789\_5331-model2.11 | -14.7661 |
|  | XP\_065066789\_5331-model2.12 | -14.7661 |
|  | XP\_065066789\_5331-model2.13 | -14.7015 |
|  | XP\_065066789\_5331-model2.14 | -14.7015 |
|  | XP\_065066789\_5331-model2.15 | -14.6582 |
|  | XP\_065066789\_5331-model2.16 | -14.6541 |
| # cluster 3 - Size: 36 |  |  |
|  | XP\_065066789\_5331-model3 | -14.8981 |
|  | XP\_065066789\_5331-model3.1 | -14.8981 |
|  | XP\_065066789\_5331-model3.2 | -14.787 |
|  | XP\_065066789\_5331-model3.3 | -14.7863 |
|  | XP\_065066789\_5331-model3.4 | -14.7863 |
|  | XP\_065066789\_5331-model3.5 | -14.7863 |
|  | XP\_065066789\_5331-model3.6 | -14.7863 |
|  | XP\_065066789\_5331-model3.7 | -14.7863 |
|  | XP\_065066789\_5331-model3.8 | -14.7863 |
|  | XP\_065066789\_5331-model3.9 | -14.7863 |
|  | XP\_065066789\_5331-model3.10 | -14.77 |
|  | XP\_065066789\_5331-model3.11 | -14.7641 |
|  | XP\_065066789\_5331-model3.12 | -14.7641 |
|  | XP\_065066789\_5331-model3.13 | -14.7503 |
|  | XP\_065066789\_5331-model3.14 | -14.7503 |
|  | XP\_065066789\_5331-model3.15 | -14.7503 |
|  | XP\_065066789\_5331-model3.16 | -14.7338 |
|  | XP\_065066789\_5331-model3.17 | -14.7311 |
|  | XP\_065066789\_5331-model3.18 | -14.7302 |
|  | XP\_065066789\_5331-model3.19 | -14.7302 |
|  | XP\_065066789\_5331-model3.20 | -14.7302 |
|  | XP\_065066789\_5331-model3.21 | -14.7302 |
|  | XP\_065066789\_5331-model3.22 | -14.7302 |
|  | XP\_065066789\_5331-model3.23 | -14.7302 |
|  | XP\_065066789\_5331-model3.24 | -14.7302 |
|  | XP\_065066789\_5331-model3.25 | -14.7302 |
|  | XP\_065066789\_5331-model3.26 | -14.7302 |
|  | XP\_065066789\_5331-model3.27 | -14.7302 |
|  | XP\_065066789\_5331-model3.28 | -14.7302 |
|  | XP\_065066789\_5331-model3.29 | -14.7302 |
|  | XP\_065066789\_5331-model3.30 | -14.7288 |
|  | XP\_065066789\_5331-model3.31 | -14.7288 |
|  | XP\_065066789\_5331-model3.32 | -14.6807 |
|  | XP\_065066789\_5331-model3.33 | -14.6807 |
|  | XP\_065066789\_5331-model3.34 | -14.6657 |
|  | XP\_065066789\_5331-model3.35 | -14.6657 |
| # cluster 4 - Size: 5 |  |  |
|  | XP\_065066789\_5331-model4 | -14.8714 |
|  | XP\_065066789\_5331-model4.1 | -14.7812 |
|  | XP\_065066789\_5331-model4.2 | -14.7812 |
|  | XP\_065066789\_5331-model4.3 | -14.77 |
|  | XP\_065066789\_5331-model4.4 | -14.7246 |
| # cluster 5 - Size: 17 |  |  |
|  | XP\_065066789\_5331-model5 | -14.8689 |
|  | XP\_065066789\_5331-model5.1 | -14.8689 |
|  | XP\_065066789\_5331-model5.2 | -14.8621 |
|  | XP\_065066789\_5331-model5.3 | -14.8603 |
|  | XP\_065066789\_5331-model5.4 | -14.8603 |
|  | XP\_065066789\_5331-model5.5 | -14.8603 |
|  | XP\_065066789\_5331-model5.6 | -14.832 |
|  | XP\_065066789\_5331-model5.7 | -14.832 |
|  | XP\_065066789\_5331-model5.8 | -14.8178 |
|  | XP\_065066789\_5331-model5.9 | -14.8178 |
|  | XP\_065066789\_5331-model5.10 | -14.8178 |
|  | XP\_065066789\_5331-model5.11 | -14.8178 |
|  | XP\_065066789\_5331-model5.12 | -14.791 |
|  | XP\_065066789\_5331-model5.13 | -14.791 |
|  | XP\_065066789\_5331-model5.14 | -14.791 |
|  | XP\_065066789\_5331-model5.15 | -14.791 |
|  | XP\_065066789\_5331-model5.16 | -14.7716 |
|  |  |  |
